# Dimethylarginine dimethylaminohydrolase I enhances tumour growth and angiogenesis

**DOI:** 10.1038/sj.bjc.6600518

**Published:** 2002-09-09

**Authors:** V Kostourou, S P Robinson, J E Cartwright, G St J Whitley

**Affiliations:** Department of Biochemistry and Immunology, St George's Hospital Medical School, London SW17 0RE, UK

**Keywords:** dimethylarginine dimethylaminohydrolase, nitric oxide, nitric oxide synthase, vascular endothelial growth factor, asymmetric dimethylarginine

## Abstract

Angiogenesis is a prerequisite for tumour progression and is highly regulated by growth factors and cytokines a number of which also stimulate the production of nitric oxide. Asymmetric dimethylarginine is an endogenous inhibitor of nitric oxide synthesis. Asymmetric dimethylarginine is metabolised by dimethylarginine dimethylaminohydrolase. To study the effect of dimethylarginine dimethylaminohydrolase on tumour growth and vascular development, the rat C6 glioma cell line was manipulated to overexpress the rat gene for dimethylarginine dimethylaminohydrolase I. Enhanced expression of dimethylarginine dimethylaminohydrolase I increased nitric oxide synthesis (as indicated by a two-fold increase in the production of cGMP), expression and secretion of vascular endothelial cell growth factor, and induced angiogenesis *in vitro*. Tumours derived from these cells grew more rapidly *in vivo* than cells with normal dimethylarginine dimethylaminohydrolase I expression. Immunohistochemical and magnetic resonance imaging measurements were consistent with increased tumour vascular development. Furthermore, dimethylarginine dimethylaminohydrolase activity was detected in a series of human tumours. This data demonstrates that dimethylarginine dimethylaminohydrolase plays a pivotal role in tumour growth and the development of the tumour vasculature by regulating the concentration of nitric oxide and altering vascular endothelial cell growth factor production.

*British Journal of Cancer* (2002) **87**, 673–680. doi:10.1038/sj.bjc.6600518 www.bjcancer.com

© 2002 Cancer Research UK

## 

Angiogenesis is the formation of new capillaries from existing blood vessels. It is a multistep process that involves dissociation of endothelial cells (EC) from adjacent pericytes, remodelling of the extracellular matrix, proliferation and migration of EC and capillary differentiation. Insight into the regulation of this process is central to our understanding of a number of biological processes whether physiological, as occurs during development, the reproductive cycle and wound healing, or pathological, as occurs during atherogenesis, diabetic retinopathy and tumour progression (Carmeliet and Jain, 2000; Folkman, 1995).

There is strong evidence to suggest that nitric oxide (NO), produced from arginine by the nitric oxide synthases (NOS), is a crucial signalling molecule and regulator of angiogenesis (Chinje and Stratford, 1997). NO enhances vascular permeability, induces extracellular matrix degradation, endothelial cell proliferation and migration (Ziche *et al*, 1994; Trachtman *et al*, 1996; Fukumura *et al*, 1997) and stimulates the expression of vascular endothelial growth factor (VEGF) while also mediating many of its angiogenic effects (Chin *et al*, 1997; Papapetropoulos *et al*, 1997). The role of NO in tumour progression is still unclear, since NO has been shown to inhibit angiogenesis (Pipili-Synetos *et al*, 1995) and NO produced by activated macrophages can be cytotoxic to tumour cells (Stuehr and Nathan, 1989). However, NO has been positively correlated with tumour grade in human gynaecological, breast, neuronal, and head and neck tumours (Thomsen *et al*, 1994; Cobbs *et al*, 1995; Gallo *et al*, 1998; Reveneau *et al*, 1999).

Regulation of NO production may therefore play an important role in the regulation of angiogenesis and consequently tumour progression. Methylated analogues of arginine such as asymmetric dimethylarginine (ADMA) and N-mono methylarginine (L-NMMA) are competitive inhibitors of NO synthesis. Free ADMA is found in plasma and urine of healthy individuals and is synthesised by the post-translational methylation of protein arginine residues and liberated upon their hydrolysis (Kakimoto and Akazawa, 1970; Fickling *et al*, 1993). The intracellular concentration of ADMA reaches levels sufficient to inhibit NO synthesis (Macallister *et al*, 1994) and can be modulated by changes in the activity of the enzyme dimethylarginine dimethylaminohydrolase (DDAH) (Ogawa *et al*, 1989; Macallister *et al*, 1996).

Two isoforms of DDAH have been reported (Leiper *et al*, 1999). DDAH I predominates in tissues that express the neuronal isoform of NOS, while DDAH II distribution mainly follows endothelial NOS (Leiper *et al*, 1999). Dysfunction of DDAH and elevated levels of its substrate ADMA have been implicated in pathological conditions including hypertension, pre-eclampsia, renal failure and atherosclerosis (Matsuoka *et al*, 1997; Holden *et al*, 1998; Miyazaki *et al*, 1999). Recently, high levels of ADMA have been correlated with impaired angiogenesis in hypercholesterolemic mice (Jang *et al*, 2000). The aim of our study was to test the hypothesis that tumour-derived ADMA plays a significant role in the regulation of tumour growth and vascular development. We demonstrate that increased DDAH expression results in decreased tumour ADMA, increased NO production, tumour growth and angiogenesis, and that these effects are mediated through changes in the expression of VEGF. We also show preliminary evidence of DDAH activity in human tumours.

## METHODS

### Cell culture and transfections

Rat C6 glioma cells (European Collection of Cell Cultures, Centre for Applied Microbiology and Research, UK) were maintained in Ham's F-10 medium containing 5 mM L-glutamine, 100 U ml^−1^ penicillin, 0.1 mg ml^−1^ streptomycin and 10% (v v^−1^) foetal calf serum. The full length rat DDAH I cDNA was generated by RT–PCR using mRNA isolated from C6 cells, forward primer: GGAGCAAGCTTCGCCACCATGGCCGGCCTCAGCCA and reverse primer: GCCTGCTCGAGTCAAGAGTCTGTCTTCTTGTTAAT and subsequently cloned into *Hind*III/*Xho*I sites in pcDNA 3.1/hygro (InVitrogen). C6 cells were transfected using the poly L-ornithine method (5×10^6^ cells per 9 cm plate; 10 μg DNA plasmid; 10 μg ml^−1^ poly L-ornithine) and selected in medium supplemented with 500 μg ml^−1^ hygromycin B (Dong *et al*, 1994).

### Animals and tumours

Female (7–8 weeks old) MF1/nu mice were injected subcutaneously in the flanks with 2×10^6^ cells in 0.1 ml PBS. Tumour growth was measured at daily intervals after anaesthetising with fluothane in 2% N_2_O, 5% O_2_. The tumour volume was calculated using the formula: tumour volume=π/6·(length×width×depth). All procedures were performed in accordance to the Home Office Scientific Procedures Act 1986.

### Western blot analysis

The protein concentration of cell lysates and tumour homogenates were determined by Bradford assay and equal amounts analysed by SDS–PAGE. DDAH expression was detected using a monoclonal antibody against rat DDAH I (Kimoto *et al*, 1995).

### DDAH activity assay

Cells were seeded on 24-well plates at a density of 1.5×10^5^ cells per well and incubated in HEPES buffered Krebs solution containing [^14^C] L-NMMA (0.8 μCi ml^−1^, specific activity 56 mCi mMol^−1^) for 1 h at 37°C. Cells were washed twice in ice-cold Krebs solution and lysed in 0.1% (w v^−1^) SDS. Human tumour biopsy specimens taken during surgical removal of the tumour, or mouse tumour tissues were homogenised in 10 mM sodium phosphate buffer pH 6.6 using a motor homogeniser. Aliquots of 500 μl were incubated with [^14^C] L-NMMA (0.4 μCi ml^−1^) for 1 h at 37°C. The formation of [^14^C] citrulline was determined as previously described (Macallister *et al*, 1996). The enzymatic activity was corrected for the protein concentration.

### HPLC analysis

Assessment of the concentration of ADMA in tumour homogenates and in culture medium was performed as previously described (Macallister *et al*, 1994).

### cGMP assay

Estimation of the production of cGMP in tumour cells was performed as previously described (Whitley, 1994).

### *In vitro* assay of endothelial cell migration

A human endothelial cell line SGHEC-7 derived from SV40 transfected human umbilical vein endothelial cells (Fickling *et al*, 1992) was cultivated on microcarrier (MC) beads for 2 days and embedded in fibrin gels (Nehls and Drenckhahn, 1995). Conditioned medium from confluent cultures mixed 1 : 1 with fresh medium was added. After 2 days incubation, digital images were acquired and the extent of migration was determined using Image Pro-Plus software (Media Cybernetics). Invasion was determined as the maximum length of invasive processes minus the radius of the bead.

### Northern blot analysis

Total RNA was prepared from cells using TRIZOL (Gibco BRL). The probe for VEGF was a 540 bp *Bam*HI-*Hind*III fragment of the human VEGF_121_ cDNA (nt 565–1012). The high identity in the cDNA sequences of human and rat or mouse VEGF ensures the crossreactivity of the probe (Shima *et al*, 1996). The probe for the 18S subunit corresponds to nucleotides 552–1429 nt of the human 18S gene.

### VEGF ELISA

Culture medium was collected from confluent 6-well plates and analysed for VEGF expression using a murine VEGF ELISA kit (R&D Systems, detection limit 3 pg ml^−1^). Tumours were excised at an early stage of development (day 15), weighed, cut in small pieces and maintained in culture medium for 4 days. VEGF secretion was measured in the culture medium. Values were correlated with the protein concentration of cells or the weight of tumours, respectively.

### Magnetic resonance imaging (MRI)

A separate cohort of size-matched D27 and C6 wild type tumours were imaged at 18 and 23 days post-passage respectively. ^1^H MR imaging was performed on a Varian spectrometer at 4.7 T using a two-turn surface coil (1 cm diameter). Mice bearing tumours derived from D27 (mean volume 1.23±0.2 cm^3^, *n*=8) or C6 (mean volume 1.25±0.2 cm^3^, *n*=8) wild type cells were anaesthetised with a single intraperitonal dose of 10 ml kg^−1^ Hypnorm/Hypnovel and placed so the tumour hung into the centre of the surface coil. The mouse core temperature was maintained at 37°C by a warm water blanket. Multi-gradient echo (MGRE) images were acquired from three transverse 1 mm thick slices through the centre of the tumour. The readout gradient was oscillated to generate a series of eight echoes, utilising a repetition time of 100 ms, initial echo of 5 ms and an echo spacing of 5 ms, resulting in sets of images with decreased R_2_* weighting. Tumour R_2_* maps for each slice were generated using all eight gradient-echo image sets by fitting an exponential model on a pixel-by-pixel basis (Robinson *et al*, 2001; Griffiths *et al*, 2002). For each slice, R_2_* was determined from a region-of-interest (ROI) encompassing the whole tumour but excluding the surrounding skin/muscle.

### Fluorescence microscopy

Mice from the same cohort as used for the MRI and bearing similarly size-matched tumours were injected intravenously with 15 mg kg^−1^ of the perfusion marker Hoechst 33342 (Sigma) 1 min prior to cervical dislocation. Tumours were rapidly excised, frozen and sections cut (10 μm). Acetone fixed sections were visualised by fluorescence microscopy. Fluorescence signals from entire tumour sections were recorded with digital images acquired under identical conditions. The area of tumour stained by Hoechst 33342 as a percentage of the total area of tumour section was calculated using anaLYSIS (Soft Image Analysis).

### Ethics

Local Ethical Committee approval was obtained for the collection of human tissues.

## RESULTS

### Overexpression DDAH in C6 glioma cells

**Figure 1:**
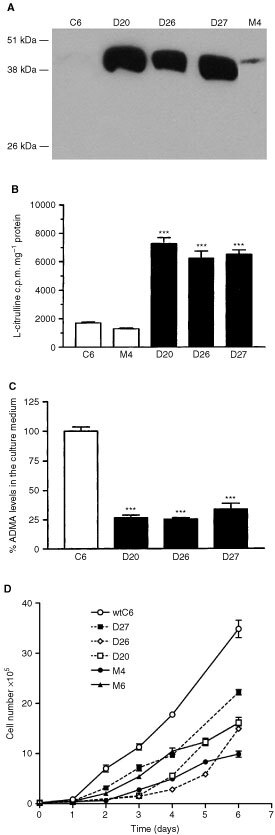
Characterisation of the stable DDAH I transfected cell lines *in vitro*. (**A**) Western blot analysis of DDAH I overexpressing cell lines (20 μg protein per lane). An increase in a band at approximately 38 kDa, indicating the presence of DDAH I was observed. (**B**) Assessment of DDAH activity in cell lysates by the conversion of [^14^C] L-NMMA to L-citrulline revealed that the D20, D26 and D27 cell lines had a four-fold increase in DDAH enzymatic activity. Results are means+s.e.m. of triplicates of three separate experiments (***P<0.001 compared to control, Student's *t*-test). (**C**) Overexpression of DDAH I reduced the concentration of ADMA by 70–75% in the culture medium as determined by HPLC analysis. Results are mean %+s.e.m. of triplicate determinations from three separate experiments (***P<0.001, Student's *t*-test). (**D**) The *in vitro* growth rate of DDAH transfected cells (dashed lines) D20, D26, D27, mock transfected cells (solid lines) M4, M6 and wild type C6 cells is shown. Results are means±s.e.m. of three experiments performed in at least duplicate.

To study the role of DDAH in tumour biology, the rat C6 glioma cell line was transfected with the pcDNA vector, containing the coding region of the rat DDAH I gene. Three stable clones, (D20, D26, D27) that constitutively express DDAH I and mock transfected clones (M lines, transfected with the empty vector as control) were analysed. Western blot analysis of D20, D26 and D27 cells revealed similarly high levels of DDAH I protein expression (Figure 1A

). Consistent with the high protein expression these cell lines displayed, on average, a four-fold increase in enzymatic activity compared to wild type C6 and control transfected M4 cells, as determined by the conversion of ^14^C radiolabelled L-NMMA to L-citrulline (Figure 1B). Since the function of DDAH is to catabolise the endogenous inhibitors of NO, ADMA and L-NMMA, the levels of ADMA in the culture medium of the overexpressing cell lines was examined. Overexpression of DDAH I was shown to reduce the levels of ADMA by 70–75% (Figure 1C). Consistent with the role of ADMA as an inhibitor of NO, the decreased levels of ADMA led to an increase in NO production as indicated by a two-fold increase in the production of cGMP (C6: 12.33±1.4, D27: 26.67±1.5, mean±s.e.mean). *In vitro* studies showed no apparent correlation between transfection with DDAH (compared to empty vector) and growth (Figure 1D). However transfected cells generally showed slower growth rates than C6 wild type cells. All three DDAH I overexpressing cell lines grew similarly to M4 and M6 (Figure 1D) and other mock transfected lines (data not shown).

### Effect of DDAH I overexpression on tumour growth *in vivo*

**Figure 2:**
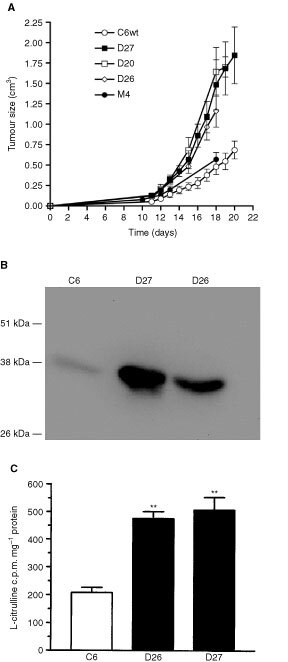
Overexpression of DDAH I increases the *in vivo* tumour growth rate. (**A**) *In vivo*, overexpression of DDAH I caused an increase in the growth rate of D20, D26 and D27 tumours compared to M4 and C6 tumours. Results are means±s.e.m. of at least two different inoculations of six mice. (**B**) Western blot analysis of tumours (100 μg protein per lane) confirmed that the overexpressing DDAH tumours had higher amounts of DDAHI protein *in vivo*. (**C**) Assessment of DDAH activity in tumour homogenates by the conversion of [^14^C] L-NMMA to L-citrulline. Tumour homogenates derived from D26 and D27 revealed a two- and a half-fold increase in their DDAH activity compared to C6 tumours. Results are means+s.e.m. of duplicates from two separate experiments (**P<0.02 compared to control, Student's *t*-test).

In contrast, when D26 and D27 cells were implanted into the flanks of nude mice their growth rate increased (doubling time: ∼2 days) compared to the wild type or the control M4 cells (doubling time: ∼4 days, Figure 2A

). In a limited number of experiments the growth of C6 tumours was allowed to continue until day 26 when they reached a similar size to that of D27 at 20 days (data not shown). Western blot analysis of tumours demonstrated higher DDAH I protein expression in D27 compared to C6 tumours (Figure 2B). Homogenates from D26 and D27 tumours displayed a 2.5-fold increase in DDAH activity (Figure 2C). The concentration of ADMA also decreased from 2.6 μg mg^−1^ protein in C6 tumours to undetectable levels in D27 tumours.

### Effect of DDAH I on tumour vascular development

**Figure 3:**
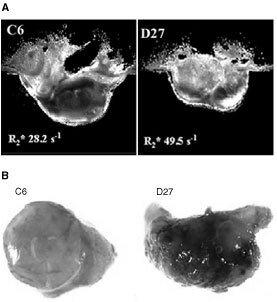
Overexpression of DDAH I affects the tumour vasculature. (**A**) Representative R_2_* maps obtained from size-matched C6 and D27 tumours, and their associated average R_2_* relaxation rates, imaged 23 days and 18 days post-passage respectively. The more intense R_2_* map (brighter image) of the D27 tumour is consistent with a high concentration of deoxyhaemoglobin and hence better vessel development. (**B**) Representative photographs of size-matched tumours derived from C6 and D27 cells. C6 and D27 tumours were excised 26 and 20 days post-implantation respectively. DDAH I overexpressing tumours are better vascularised compared to wild type or control tumours.

Non-invasive Magnetic Resonance Imaging (MRI) was employed to assess the blood vessel development *in vivo*. Deoxyhaemoglobin is paramagnetic and creates magnetic susceptibility perturbations around blood vessels, increasing the MRI transverse relaxation time R_2_*. Multi-gradient recalled echo (MGRE) MR images allow the relaxation rate R_2_* to be quantified, which is directly proportional to the tissue content of deoxyhaemoglobin and hence a sensitive index of tissue vascularity (Robinson *et al*, 2001; Griffiths *et al*, 2002). The average R_2_* of D27 and C6 tumours was 65.6±5 s^−1^ and 42.5±4 s^−1^ respectively. The significantly faster R_2_* displayed by the D27 tumours compared to the wild type (Student's *t*-test, *P*<0.001) indicates a higher concentration of deoxyhaemoglobin and increased tumour vascularisation (Figure 3A

). In addition to the higher growth rate, tumours derived from the D27 cells were shown upon excision to bleed profusely and to be more vascularised compared to wild type tumours (Figure 3B).

### DDAH I expression results in increased tumour perfusion

The effect of DDAH I on tumour perfusion was examined using the fluorescent marker Hoechst 33342 (Smith *et al*, 1988). This is a nuclear dye that, upon injection, stains endothelial cells and cells immediately adjacent to tumour blood vessels. Since the time of experiment was limited to 1 min to avoid diffusion of the dye, Hoechst 33342 marked only the functionally perfused vessels. Fluorescence microscopy showed that the extent of the staining is greater in D27 tumours compared to wild type tumours indicating better tumour perfusion and increased number of functional vessels (C6: 5.1%±0.5, D27: 9%±0.5, mean±s.e.m, *n*=4).

### DDAH I induces *in vitro* vascular endothelial cell migration

**Figure 4:**
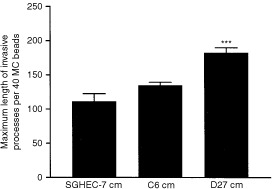
Conditioned medium from DDAH I overexpressing cells induces endothelial cell migration *in vitro*. A human endothelial cell line SGHEC-7 was grown on beads in three dimensional fibrin gels and incubated with conditioned medium (cm) from confluent SGHEC-7, D27 or C6 cells. Quantitative analysis of invasive process formation was expressed as the average of the maximum length of process minus the radius of the bead per 40 MC beads. Results are means+s.e.m. of 40 individual measurements in three separate experiments (***P<0.001 compared to control, Student's *t*-test).

The role of DDAH I on vessel development was examined further using an *in vitro* assay of endothelial migration. Human endothelial cells, SGHEC-7, grown on microcarrier (MC) beads and embedded in fibrin gels were incubated with conditioned medium from confluent cultures of C6, D27 or SGHEC-7 cells and the formation of invasive processes was analysed over a period of 2 days. Invasion of endothelial cells into the fibrin gel was used as an indicator of the angiogenic potential of factors produced by the D27 cells. The average of the maximum length of the process minus the radius of the bead from 40 MC beads was determined. Statistical analysis showed that conditioned medium from D27 cells significantly stimulated the migration of SGHEC-7 cells into the fibrin gel compared to medium from C6 or SGHEC-7 cells (Figure 4

).

### DDAH I expression increases the production of VEGF

**Figure 5:**
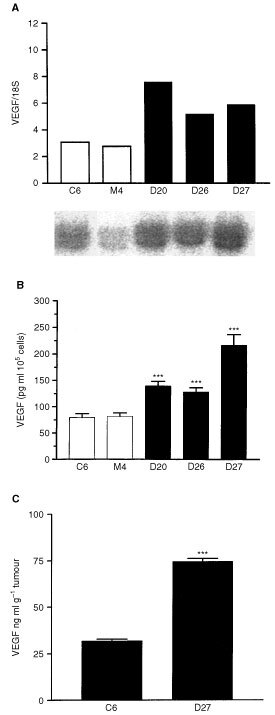
Overexpression of DDAH I results in upregulation of VEGF. (**A**) VEGF mRNA expression in the D27, D26, D20, M4 and parent C6 cell lines assessed by Northern blot analysis. RNA loading was corrected according to the levels of the 18S ribosomal subunit. The signal intensity was quantified by densitometry and expressed as the ratio of VEGF/18S. The lower panel shows a representative Northern blot analysis of VEGF expression in cell lysates. (**B**) VEGF expression in the culture medium of D27, D26, D20, M4 and parent C6 cell lines assessed by ELISA. Results are means+s.e.m. of duplicates of three individual experiments (***P<0.001 compared to control, Student's *t*-test). (**C**) VEGF secretion in the culture medium of D27 and C6 derived tumours were assessed by ELISA. Results are means+s.e.m. of triplicates of three tumours respectively (***P<0.001, Student's *t*-test).

Since it has previously been suggested that NO can mediate some of the angiogenic effects of VEGF, we examined whether the effects of DDAH and ADMA on tumour angiogenesis were mediated through changes in VEGF. *In vitro* analysis of VEGF mRNA displayed a two-fold increase in D20, D26, D27 compared to control M4 and wild type C6 cells as determined by Northern blot analysis (Figure 5A

). This correlated with an average two-fold increase in VEGF secretion in the culture medium as determined by ELISA (Figure 5B). *Ex vivo* analysis of VEGF secretion displayed a two-fold increase in D27 compared to C6 tumour culture supernatant (Figure 5C).

### DDAH I activity in human tumours

**Figure 6:**
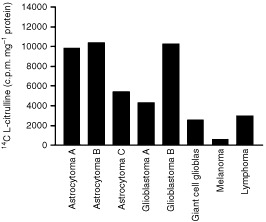
Presence of DDAH activity in human tumours. DDAH activity in tumour homogenates derived from eight individual human tumour biopsy specimens taken during surgical removal of the tumour. DDAH activity was assessed by the conversion of [^14^C] L-NMMA to L-citrulline. DDAH activity varied in the different tumour types.

DDAH activity was detected in eight human tumours, including those from the brain (Figure 6

).

## DISCUSSION

Tumour angiogenesis is driven by the requirement of the tumour for nutrients and oxygen. Angiogenesis involves the coordination of a number of independent events including the production of proteases and angiogenic factors that have both a chemotactic and mitogenic effect on endothelial cells. We demonstrate a novel mechanism by which a reduction in tumour ADMA, as a result of increased DDAH activity, stimulates tumour growth and angiogenesis through increased NO and VEGF expression.

Regulation of the production of NO through DDAH activity is important since NO can have pleiotropic effects on diverse aspects of tumour biology. There is positive correlation between NOS activity and tumour grade in human breast, gynaecological, head, neck and neuronal tumours (Thomsen *et al*, 1994; Cobbs *et al*, 1995; Gallo *et al*, 1998; Reveneau *et al*, 1999) and NOS inhibitors have been shown to decrease tumour growth (Thomsen *et al*, 1997). However, the role of tumour-derived NO is still unclear since overexpression of iNOS in tumour cells can promote or inhibit tumour growth and metastasis (Dong *et al*, 1994; Jenkins *et al*, 1995; Xie *et al*, 1995).

Increased malignancy of human primary brain tumours and the formation of metastasis is associated with increased neovascularisation. Astrocytomas represent one of the most vascularised human neoplasms and the progression from low to high grade is characterised by an increase in neovascularisation, focal necrosis and cellular proliferation. Tumours derived from the rat C6 glioma cell line are histologically classified as astrocytomas and represent a well-established model of human glioblastomas (Barth, 1998). C6 glioma cells express both eNOS and iNOS (Simmons and Murphy, 1993; Barna *et al*, 1996).

The result of increased DDAH I activity in C6 cells and the concomitant fall in the concentration of ADMA led to a two-fold increase in NO synthesis as observed by increased cGMP production. However this did not alter the *in vitro* growth properties of the glioma cell lines. The *in vivo* growth rate of D20, D26 and D27 cells was significantly increased compared to the wild type or the control M4 cells. Our data suggest that DDAH/ADMA pathway does not have a direct effect on the cell cycle or cell proliferation but indirectly affects the tumour growth, possibly by interfering with the tumour vessel development.

To establish a role for ADMA and DDAH in tumour angiogenesis *in vivo,* MGRE-MRI was used as a non-invasive assay of blood vessel development of D27 and wild type C6 tumours, followed by gross histology and fluorescence microscopy using the nuclear dye Hoechst 33342. It is clear from these experiments that DDAH I expression leads to higher blood volume, better tumour perfusion and increased number of functional vessels. We can therefore speculate that the increased growth observed in D27 tumours is dependent on increased blood vessel development.

Sprouting of endothelial cells from pre-existing vessels, the invasion into the surrounding tissue and the reorganisation into new capillary structures are important steps in vessel development. DDAH I expression and ADMA concentration does not affect the proliferation rate of endothelial cells (V Kostourou, unpublished observations). Therefore, a possible role of DDAH I in vessel development could be the facilitation of the endothelial cell invasion. In support of this, conditioned medium from D27 cells significantly stimulated the migration of SGHEC-7 cells into the fibrin gel compared to medium from C6 or SGHEC-7 cells. These results demonstrate that DDAH activity and the intracellular concentration of ADMA regulate the release of an angiogenic factor or factors *in vitro* that affect the invasive properties of the endothelial cells.

Vessel growth and development is under the control of a number of factors the most potent being VEGF. VEGF is a largely endothelial cell-specific mitogen that is induced by hypoxia. It promotes angiogenesis, blood vessel permeability and maintains tumour vessel integrity (Shweiki *et al*, 1992; Yancopoulos *et al*, 2000). Increased VEGF expression is related to neovascularisation and tumour progression (Takahashi *et al*, 1995; Zhang *et al*, 1995) while inhibition of VEGF or its receptors inhibits tumour growth *in vivo* (Kim *et al*, 1993; Saleh *et al*, 1996). Since NO may function as an upstream regulator of expression as well as a downstream effector of the action of VEGF, we examined whether the effects of DDAH and ADMA on tumour angiogenesis were mediated through changes in VEGF. *In vitro* analysis of VEGF mRNA production as well as VEGF protein secretion in the culture medium demonstrated that DDAH I expression stimulates VEGF production. The increase in VEGF production was similar to that induced by hypoxia in the same cells (V Kostourou, unpublished observations).

Our findings indicate that human tumours exhibit DDAH activity. Although this study is limited in number it does suggest that human brain tumours express particularly high levels of DDAH activity. Increased NO production has also been reported in human CNS tumours (Cobbs *et al*, 1995). Our experimental data demonstrates that DDAH I can promote tumour growth and increase tumour perfusion. Since tumours with a high grade of malignancy are better perfused and vascularised it is interesting to speculate that DDAH I could facilitate tumour progression and correlate with tumour malignancy. The relationship between DDAH activity and tumour grade is currently under investigation.

In summary, this study shows that increased DDAH expression results in decreased tumour ADMA, increased NO and VEGF production, vascularisation and tumour growth. Our results provide the first demonstration of the importance of the ADMA/DDAH pathway in the regulation of vessel development. It also has important implications for the regulation of angiogenesis in other pathological and physiological circumstances in which VEGF expression or NO production is involved. The fact that tumour development is so dramatically altered by regulating this pathway adds a novel level of complexity to a system where the precise balance between pro- and anti-angiogenic factors is of key importance. Therapeutic interventions in oncology using the DDAH/ADMA pathway are therefore plausible.
